# Publisher Correction: Capturing variation impact on molecular interactions in the IMEx Consortium mutations data set

**DOI:** 10.1038/s41467-019-08814-w

**Published:** 2019-03-04

**Authors:** IMEx Consortium contributing authors, J. Khadake, B. Meldal, S. Panni, D. Thorneycroft, K. van Roey, S. Abbani, L. Salwinski, M. Pellegrini, M. Iannuccelli, L. Licata, G. Cesareni, B. Roechert, A. Bridge, M. G. Ammari, F. McCarthy, F. Broackes-Carter, N. H. Campbell, A. N. Melidoni, M. Rodriguez-Lopez, R. C. Lovering, S. Jagannathan, C. Chen, D. J. Lynn, S. Ricard-Blum, U. Mahadevan, A. Raghunath, N. del-Toro, M. Duesbury, M. Koch, L. Perfetto, A. Shrivastava, D. Ochoa, O. Wagih, J. Piñero, M. Kotlyar, C. Pastrello, P. Beltrao, L. I. Furlong, I. Jurisica, H. Hermjakob, S. Orchard, P. Porras

**Affiliations:** 1 European Bioinformatics Institute (EMBL-EBI), European Molecular Biology Laboratory, Wellcome Genome Campus, Hinxton, CB10 1SD UK; 2 Novartis Institutes for BioMedical Research (NIBR), Maulbeerstrasse 66, 4058 Basel, Canton of Basel-Stadt Switzerland; 3 Deep Genomics, MaRS Centre, 661 University Ave, Suite 480, Toronto, ON M5G 1M1 Canada; 4 Research Programme on Biomedical Informatics (GRIB), Department of Experimental and Health Sciences (DCEXS), Hospital del Mar Medical Research Institute (IMIM), Universitat Pompeu Fabra (UPF), 08003 Barcelona, Spain; 5 Krembil Research Institute, Data Science Discovery Centre for Chronic Diseases, University Health Network, 5KD-407, 60 Leonard Avenue, Toronto, ON M5T 0S8 Canada; 6 Departments of Medical Biophysics and Computer Science, University of Toronto, Toronto, M4B 1B5 Canada; 7 State Key Laboratory of Proteomics, Beijing Proteome Research Center, National Center for Protein Sciences, Beijing Institute of Life Omics, 102206 Beijing, China; 8 DiBEST Department, University of Calabria, Via Pietro Bucci, Rende (CS), 87036 Italy; 9 Los Angeles Department of Energy Institute for Genomics and Proteomics, University of California, Los Angeles, CA 90001 USA; 10 Department of Biology, University of Rome Tor Vergata, Via della Ricerca Scientifica, 00118 Rome, Italy; 11 Swiss-Prot group, SIB Swiss Institute of Bioinformatics, CMU 1, Rue Michel Servet, 1211 Geneva 4, Switzerland; 12 School of Animal and Comparative Biomedical Sciences, University of Arizona, Tucson, AZ 85721 USA; 13 Functional Gene Annotation, Institute of Cardiovascular Science, University College London, London, 56273 UK; 14 Mechanobiology Institute, National University of Singapore, T-Lab #05-01, 5A Engineering Drive 1, Singapore, 117411 Singapore; 15 Centre for Microbial Diseases and Immunity Research, University of British Columbia, Lower Mall, Vancouver, 2259 Canada; 16 South Australian Health and Medical Research Institute, EMBL Australia Group, North Terrace, Adelaide, SA 5001 Australia; 17 The College of Medicine and Public Health, Flinders University, Bedford Park, SA 5042 Australia; 18 Univ. Lyon, ICBMS, UMR 5246 CNRS, Université Lyon 1, Villeurbanne Cedex, 69622 Lyon, France; 19 Molecular Connections Pvt. Ltd., Kandala Mansions, 2/2 Kariappa Road, Basavangudi, Bangalore, 560004 India

Correction to: *Nature Communications* 10.1038/s41467-018-07709-6; published online 02 January 2019

In the original HTML version of this Article, the order of authors within the author list was incorrect. The IMEx Consortium contributing authors were incorrectly listed as the last author and should have been listed as the first author. This error has been corrected in the HTML version of the Article; the PDF version was correct at the time of publication.

