# Supplementary material for: Capturing variation impact on molecular interactions in the IMEx Consortium mutations data set
Source: Nat Commun. 2019 Jan 2;10:10. doi: 10.1038/s41467-018-07709-6 (PMC6315030; doi:10.1038/s41467-018-07709-6)
Supplement: Supplementary file 1 — Supplementary Information [file 41467_2018_7709_MOESM1_ESM.pdf]

Supplementary information

Initial re-curation of mutation data in IMEx

The data described in this paper has incrementally been made available to the research community since 2007 in PSI-MI XML2.5 files, but the capture of mutant data is incomplete in this format. Although the coordinates data is captured and a Controlled Vocabulary (CV) term describes the effect, the actual amino acid change is not registered. This issue has been addressed and corrected in the recently released PSI-MI XML3.0. In order to populate the replacement amino acid information, initial versions of our automated quality control pipeline were repeatedly applied over the entire data set, enhancing over 75% of the annotations. In addition to this, a significant number of entries have been manually re-curated, when there were too many changes in the reference sequence to allow automatic fixes. The full re-curation effort allowed to recover over 90% of existing annotations. The 2,310 annotations for which it was not possible to determine the exact amino acid change are excluded from the data set but kept in IMEx records as 'undefined mutation' and are scheduled for eventual re-curation.

Automated quality control pipeline for mutation entries in IMEx

UniProtKB entries change over time and accession numbers are obsoleted, merged and de-merged. The underlying protein sequences are often updated and positional features need re-mapping to the new sequence. In order to keep the data correctly annotated and in sync with current proteome builds as provided by UniProtKB, we have developed a 'mutations update' pipeline that is run before every IntAct release. This pipeline is run immediately after the 'protein update' pipeline, which keeps proteins in IntAct in sync with the UniProtKB entries they reference. Both pipelines are able to deal with most sequence changes, with difficult cases being referred to a human curator for manual checking. A diagram of how the 'mutations update' pipeline works can be seen in Supplementary Figure 1.

Every participant feature of type 'mutation (MI:0118)' or its children is checked using this pipeline before an IntAct release. After a number of preliminary sanity checks, mutation features are then checked for range consistency, concordance between the

HGVS-compliant short label and the ‘resulting sequence’ field and correct use of amino acid code. If any problems are found or there are changes due to an update in the reference UniProtKB entry, an annotation is added at the feature level and a new short label is proposed, if possible. All corrected entries undergo manual check and correction, if needed. Annotations that cannot be fixed are labelled as ‘unspecified mutation’ and discarded from the dataset using the special annotation ‘no-mutation-export’. We retain a record of previous annotations in case they can be fixed in the future. During the design phase of this pipeline and the first bulk updates of historical mutation annotations, approximately 16,000 annotations were updated, with 1,400 requiring manual intervention. Since the introduction of the pipeline into routine IntAct production process in September 2016 and up to June 2018, 1,090 mutation annotations were automatically updated, with a further 634 requiring manual intervention.

Supplementary figures

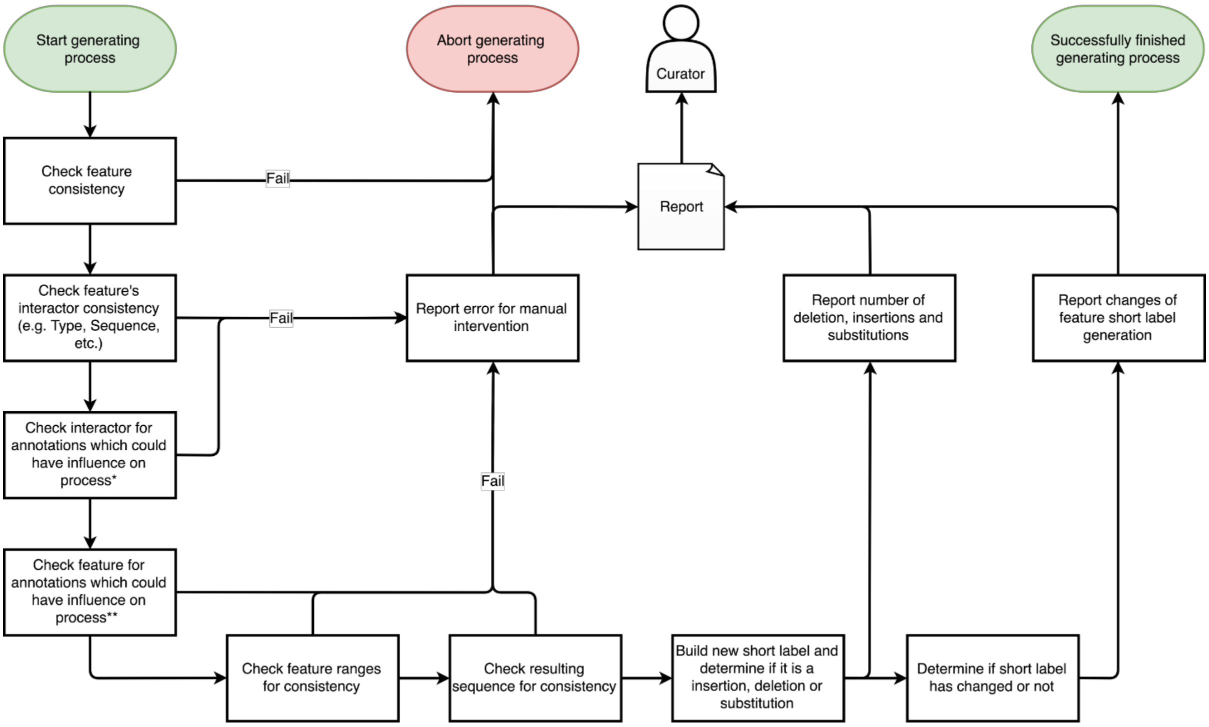

Supplementary Figure 1. 'Mutation update' pipeline diagram

* Interactor annotations: An interactor can hold several different annotations, which help us to determine its characteristics, such as if it can be kept in synch with a referenced entry in UniProtKB. If an interactor is marked with the annotation 'no-uniprot-update', it means it is not possible to keep it in sync with UniProtKB and we do not consider it for the short label generation process. ** Feature annotations: A feature can hold several different annotations, which provides context for the quality control procedure. If a feature is marked with the annotation 'no-mutation-export', we do not consider it for the short label generation. When the feature is annotated as 'no-mutation-update', we still check its consistency, but do not calculate a new short label for it.

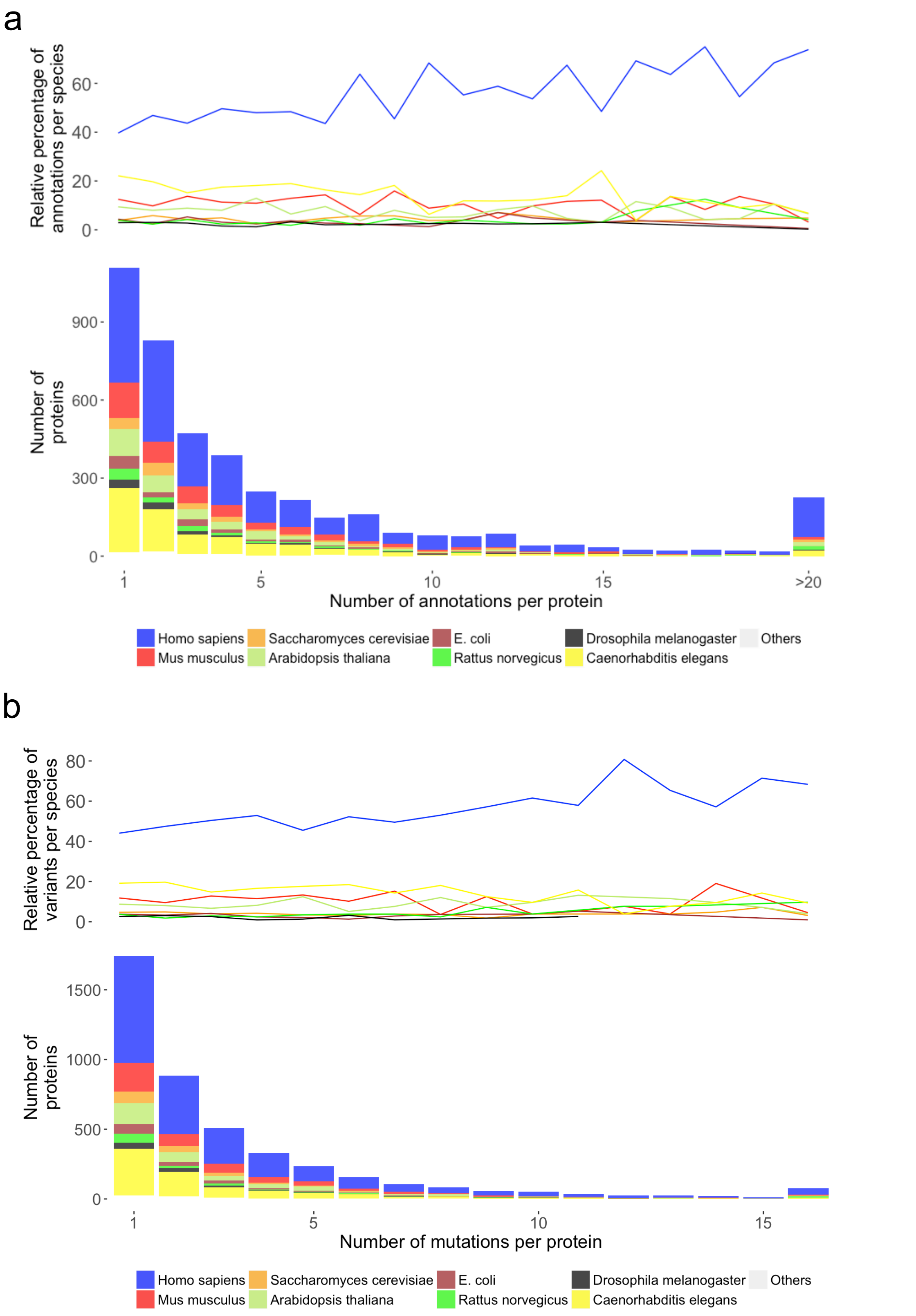

Supplementary Figure 2. Annotation depth by species

a: Relative percentage of mutation annotations per species (upper panel), along with distribution of proteins by number of annotations and species (lower panel); b: Relative percentage of variants per species (upper panel), along with distribution of proteins by number of variants and species (lower panel).

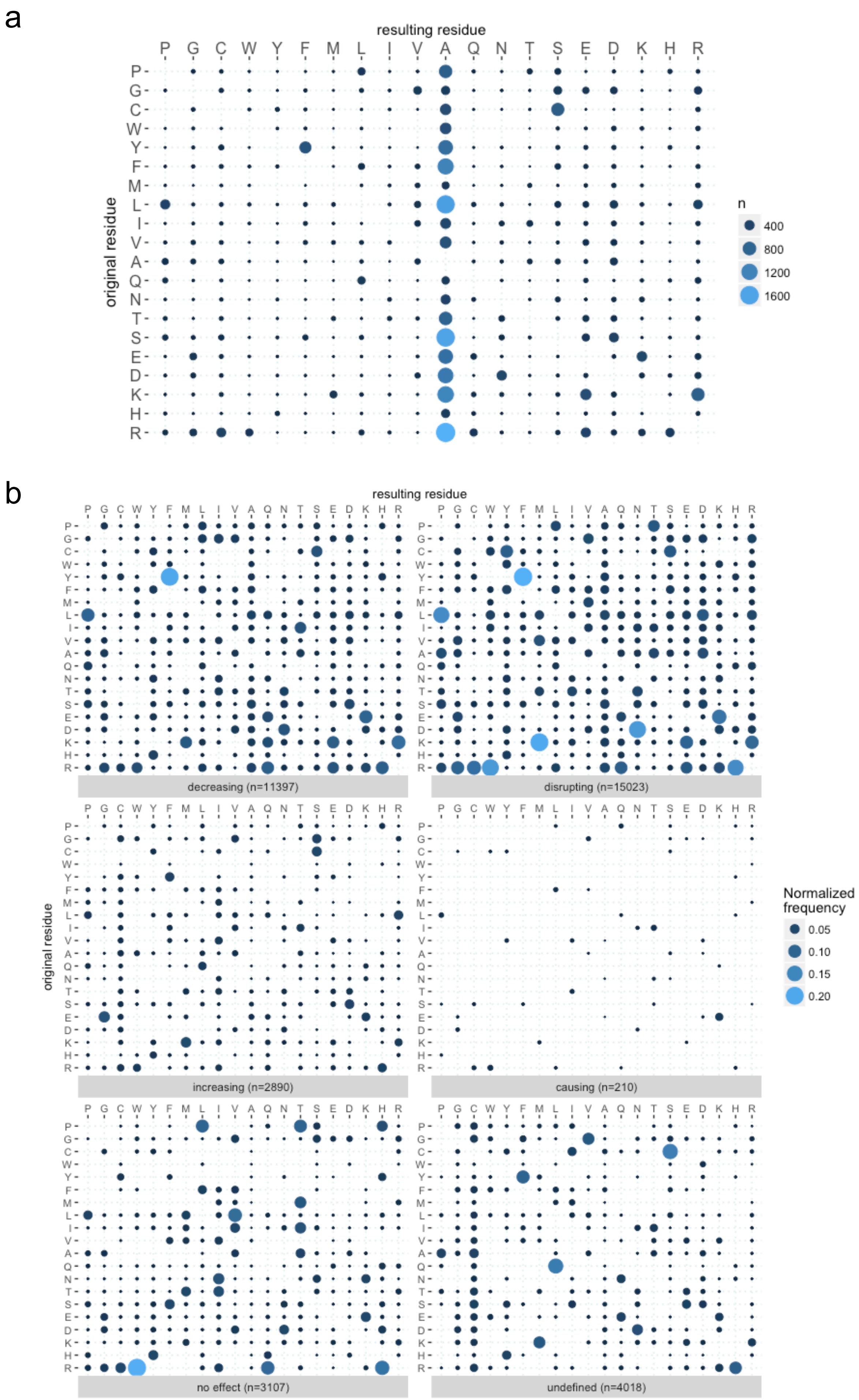
a
resulting residue
P G C W Y F M L I V A Q N T S E D K H R
original residue
P G C W Y F M L I V A Q N T S E D K H R
n
400
800
1200
1600
b
resulting residue
original residue
P G C W Y F M L I V A Q N T S E D K H R
decreasing (n=11397)
disrupting (n=15023)
increasing (n=2890)
causing (n=210)
no effect (n=3107)
undefined (n=4018)
Normalized frequency
0.05
0.10
0.15
0.20

Supplementary Figure 3. Amino acid replacements frequencies

a: Detailed matrix plot for amino acid replacement frequencies over the whole data set; b: Detailed matrix plot for normalized replacement frequencies by mutation effect. Substitutions with non-standard amino acids and deletions are not shown for simplicity.

a

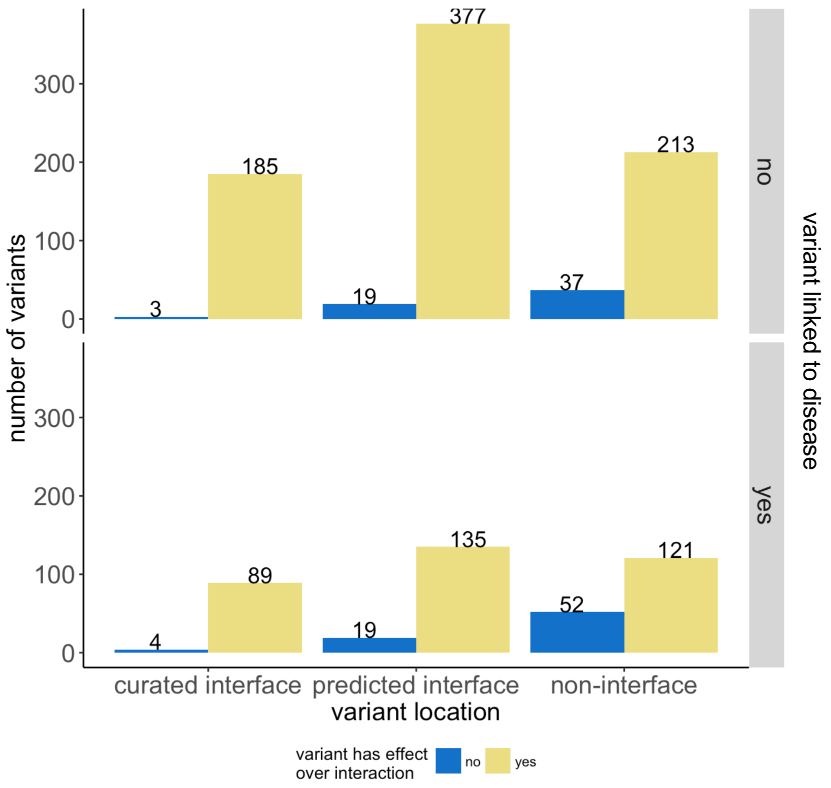

b

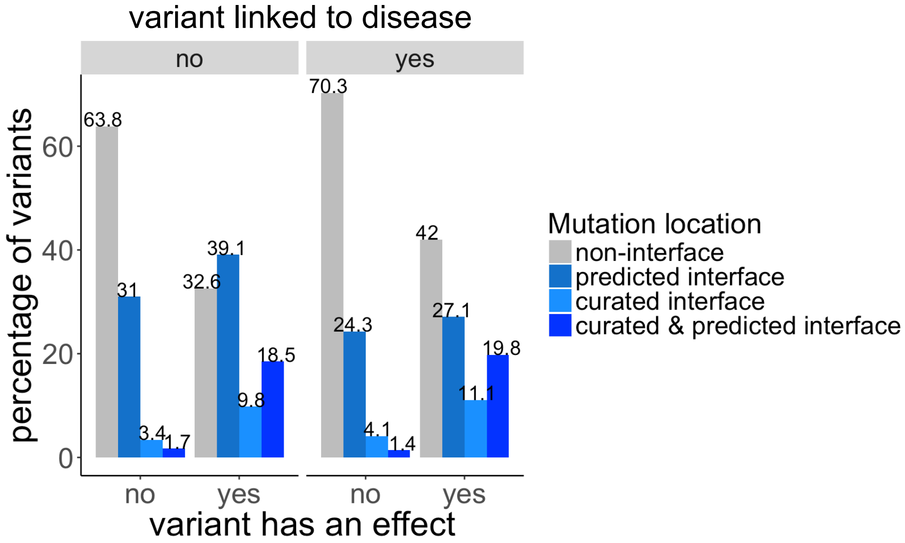

Supplementary Figure 4. Computational annotations and the IMEx mutations data set (related to figures 4g and 4h)

a: Number of variants located in binding interfaces (curated and predicted), by effect;

b: Normalized frequencies of variants reporting effects over interactions and their localization in binding interfaces.

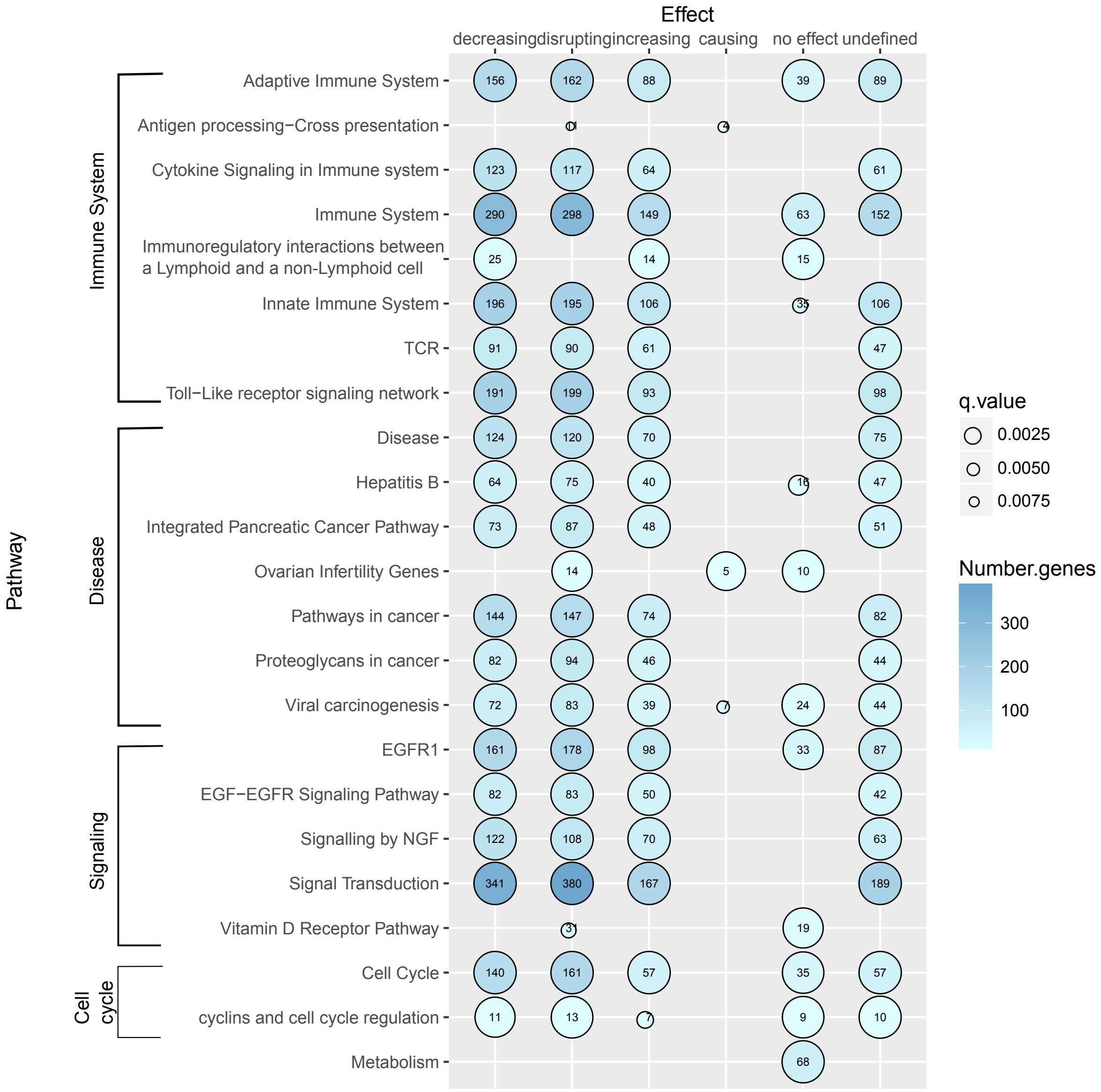

Supplementary Figure 5. PathDIP annotation analysis of mutation-influenced interactions (related to figure 6)

q-value (log scale) for top 10 pathways in each set, grouped by topic where possible. Analysis in this figure was performed considering human proteins only.

a

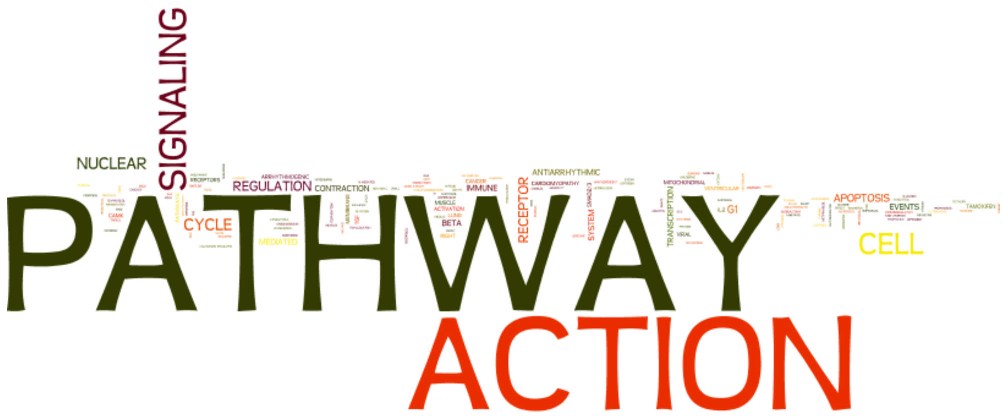

b

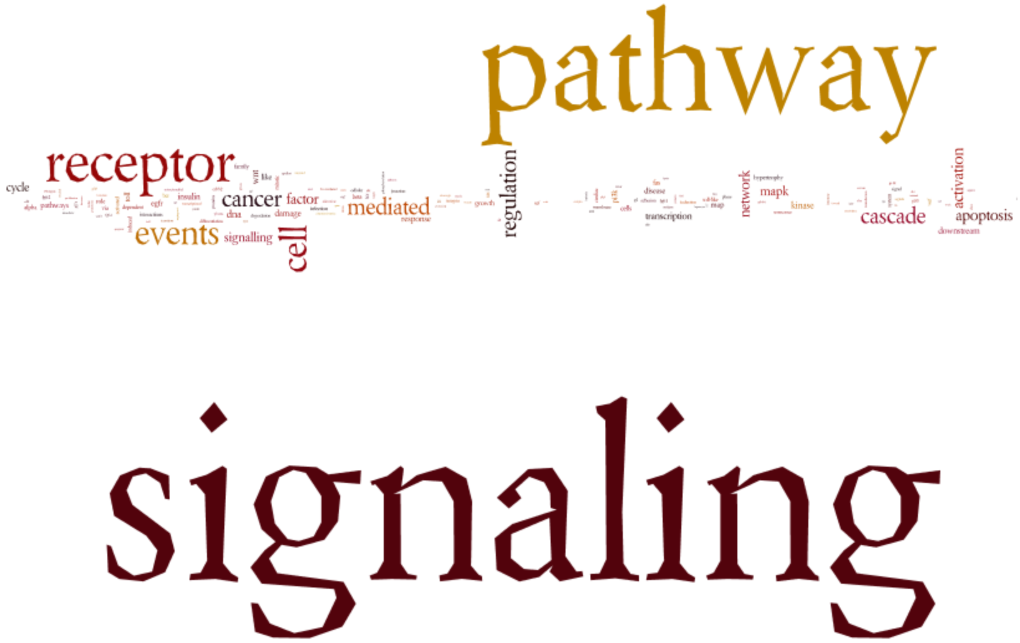

Supplementary Figure 6. Original word clouds for figure 6c

a. Original word cloud for the ‘no effect’ enrichment analysis results; b: Original word cloud for the ‘common mutated’ enrichment analysis results.

Supplementary tables

Supplementary Table 1.

Feature AC	Feature short label	Feature range(s)	Original sequence	Resulting sequence	Feature type	Feature annotations	Affected protein AC	Affected protein symbol	Affected protein full name	Affected protein organism	Interaction participants	PubMedID	Figure legend	Interaction AC
EBI-10828532	p.Arg725Glu	725-725	R	E	mutation(MI:0118)	MI:0612 (comment): Disrupts association with VPS33A and decreases association of VPS18	uniprotkb:Q9H269	VPS16	Vacuolar protein sorting-associated protein 16 homolog, hVPS16	9606 - Homo sapiens	uniprotkb:Q9P253(protein(MI:0326), 9606 - Homo sapiens);uniprotkb:Q9H269(protein(MI:0326), 9606 - Homo sapiens)	25783203	2D	EBI-10828524
EBI-985220	p.Ile114Gly	114-114	I	G	mutation increasing(MI:0382)		uniprotkb:P61316	lolA	Outer-membrane lipoprotein carrier protein (P20)	83333 - Escherichia coli (strain K12)	uniprotkb:P61316(protein(MI:0326), 83333 - Escherichia coli (strain K12)); uniprotkb:P69776(protein(MI:0326), 83333 - Escherichia coli (strain K12))	16354671	6	EBI-985197
EBI-4370347	p.[Asn31His;Ala60Val]	31-31	D	H	mutation increasing(MI:0382)	- (kd): 11e-9M	uniprotkb:P10415-1	BCL2	Apoptosis regulator Bcl-2	9606 - Homo sapiens	uniprotkb:Q13794(protein(MI:0326), 9606 - Homo sapiens);uniprotkb:P10415-1(protein(MI:0326), 9606 - Homo sapiens)	21454712	1b, 1d, s1b, S1c and 1e	EBI-4370302
EBI-4370347	p.[Asn31His;Ala60Val]	60-60	A	V	mutation increasing(MI:0382)	- (kd): 11e-9M	uniprotkb:P10415-1	BCL2	Apoptosis regulator Bcl-2	9606 - Homo sapiens	uniprotkb:Q13794(protein(MI:0326), 9606 - Homo sapiens);uniprotkb:P10415-1(protein(MI:0326), 9606 - Homo sapiens)	21454712	1b, 1d, s1b, S1c and 1e	EBI-4370302
EBI-10688294	p.Thr2Ala	2-2	T	A	mutation decreasing rate(MI:1130)		uniprotkb:P61020	RAB5B	Ras-related protein Rab-5B	9606 - Homo sapiens	uniprotkb:P61020(protein(MI:0326), 9606 - Homo sapiens);uniprotkb:Q5S007(protein(MI:0326), 9606 - Homo sapiens)	25605758	1B	EBI-10688276
EBI-9635600	p.Cys_Ser215-216Ala_Ala	215-216	CS	AA	mutation disrupting(MI:0573)		uniprotkb:P18031	PTPN1	Tyrosine-protein phosphatase non-receptor type 1, EC 3.1.3.48	9606 - Homo sapiens	uniprotkb:P18031(protein(MI:0326), 9606 - Homo sapiens);uniprotkb:P10599(protein(MI:0326), 9606 - Homo sapiens)	24976139	f5	EBI-9635586

Representative example of the data provided in the IMEx mutations data set, fully expanded. Columns describing the mutation and its effect (‘Feature AC’ to ‘Feature annotations’) are described under Table 1. Affected proteins are identified with UniProt accessions (‘Affected protein AC’), with additional details provided for ease of use (‘Affected protein symbol’, ‘Affected protein full name’, ‘Affected protein organism’). Basic reference details for the affected interaction are also featured (‘Interaction participants’, ‘PubMedID’, ‘Figure legend’, ‘Interaction AC’). Full description of the affected interaction can be obtained by searching the IntAct website with ‘Interaction AC’.
