# Supplementary material for: Capturing variation impact on molecular interactions in the IMEx Consortium mutations data set
Source: Nat Commun. 2019 Jan 2;10:10. doi: 10.1038/s41467-018-07709-6 (PMC6315030; doi:10.1038/s41467-018-07709-6)
Supplement: Supplementary file 3 — Description of Additional Supplementary Files [file 41467_2018_7709_MOESM3_ESM.pdf]

Description of Additional Supplementary files:

- *Supplementary Data 1: Comparison of common records in the IMEx mutations data set, UniProt mutagenesis annotations and the SKEMPI 2.0 database. Effect descriptions have been simplified to facilitate comparison, with deleterious and increasing/causing effects being qualified as ‘loss’ and ‘gain’, respectively.*
- *Supplementary Data 2: Annotations with conflicting effects reported in the IMEx mutations data set.*
- *Supplementary Data 3: IMEx data set mutation annotations associated with disease according to UniProtKB and DisGeNET.*
- *Supplementary Data 4: Pathways enriched in PathDIP analysis for each effect group (refers to figure 6c and Supplementary Figure 5).*
- *Supplementary Data 5: Pathway sets used for word clouds (refers to figure 6c and Supplementary Figure 6).*
